# Rare radiological manifestation of enchondromatosis in children: Columnar pattern: A retrospective cohort study

**DOI:** 10.1097/MD.0000000000039106

**Published:** 2024-07-26

**Authors:** Ahmet Salduz, Serkan Bayram, Mesut Bulakci

**Affiliations:** a Department of Orthopaedics and Traumatology, Istanbul University, Istanbul Faculty of Medicine, Istanbul, Turkey; b Department of Radiology, Istanbul University, Istanbul Faculty of Medicine, Istanbul, Turkey.

**Keywords:** columnar pattern, enchondroma, musculoskeletal system, radiography

## Abstract

The columnar cartilage pattern is characterized by parallel aligned cartilage tissue columns related to the physis without matrix calcification separated by the surrounding osseous tissue. Usually, it is seen in patients with multiple enchondromas. The objective of this study was to elucidate the clinical and radiological features of this rare radiological pattern in the physis, which remains unfamiliar to most physician. We retrospectively evaluated the clinical features and imaging findings of 15 patients (9 men and 6 women) who have a columnar pattern with varied spectrum of enchondromatosis. On X-ray and computed tomography (CT) examination, all these lesions were seen as vertical or oblique oriented tubular zones, which have relatively low radiologic density compared with normal bone. The lesions have similar signal characteristics relative to epiphyseal cartilage plates, on T1W and T2W magnetic resonance images. Columnar pattern was observed in different appearances from one single column in one physis to multiple columns in multiple physis. The mean follow-up was 62 months (range: 36–96 months). The mean age was 9.7 (range: 4–14) years at the initial admission. Eight patients had 3 or less affected physis. Five patients had only one affected physis. We defined these patients’ group who had up to 3 affected physis as “limited enchondromatosis with columnar pattern (LE-CP).” We observed that most of the columnar cartilage was turning into the normal bone via endochondral ossification. Based on our observations, the columnar pattern is a rare manifestation of the enchondromas. Columnar pattern, along with the related physis, acts as a normal endochondral ossification process, and surgery is not necessary unless there is a risk of fracture or severe deformity. Further awareness of this unique subset of patients may improve our understanding of the disease and lead to better patient outcomes. We have modified non-hereditarily enchondromatosis into 2 categories: limited enchondromatosis with the columnar pattern and multiple enchondromatosis. We believe that LE-CM reflects a developmental anomaly of the physis rather than a true neoplasia, and it acts as a normal endochondral ossification process. Level IV (case series)

## 1. Introduction

The etiology of the benign cartilage lesions is implicated by the epiphyseal plate.^[1–4]^ Enchondromas are typically located centrally of the metaphyseal area of the tubular bone. A typical appearance on radiologic images is the presence of cartilage islands separated by normal bone marrow and host bone trabeculae in the metaphyseal area of the tubular bones. These cartilage islands can contain calcifications of various shapes, such as fine linear, punctate, and popcorn calcification. It can arise in every bone, age, and gender.^[5–8]^ Although half of the cases in long tubular bones are first discovered incidentally on radiological images in the third or fourth decade of life, phalangeal enchondromas are almost always discovered with a pathologic fracture. Recent studies show some evidence regarding etiology of the enchondromas, which is physis growth abnormality related to the PTHR1 and IDH1/2 gene mutation.^[9–11]^ Enchondromatosis or Ollier disease is a rare congenital disorder characterized by multiple enchondromas that may present with shortening, enlargement, and various deformities of the affected bones.^[3,5–7,9,10]^

The columnar pattern represents an atypical appearance among enchondromas, showing distinct radiological features that differ from the typical appearance of this condition. Prominent characteristics include the presence of non-calcified, vertical, or oblique oriented cartilage columns originating from the physis.^[12]^ Additionally, its clinical behavior deviates from typical enchondromas, as these lesions often demonstrate a transition to normal bone in the majority of cases upon physis closure.

Etiology of the enchondroma or enchondromatosis is based on Jaffe postulate. It proposes that the origin of solitary and multiple enchondromas is linked to embryonic rests or “nidi” emerging from the epiphyseal plate. These formations are believed to be responsible for the growth of these tumors, eventually becoming displaced in the metaphysis. The gross pathologic, radiologic, and histologic features may vary based on the size and number of “nidi” formation. The location of the lesions specifies the histologic features of chondromas. Hypocellular bland histology is seen in proximally located and particularly isolated lesions of long bones. However, hypercellularity may be found in enchondromas located in the hand, juxtacortical chondromas, or multiple enchondromatosis.^[13]^

**Figure 1.**
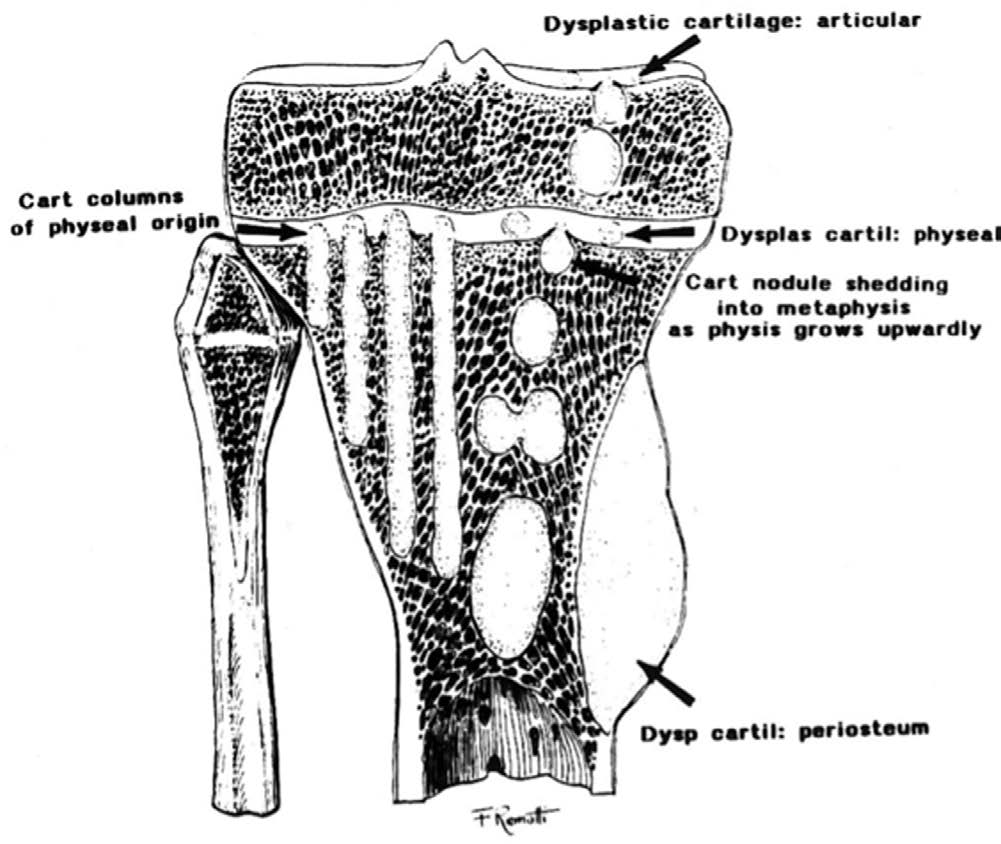
This illustration was cited from the study by Brien et al^[1]^ and provides a clear demonstration of the dysplastic cartilage of the physis in the various presentations of Ollier diseases.

Mirra et al expanded Jaffe postulate based on specimens from young and skeletally immature patients with multiple enchondromas. They identified 2 histological forms of dysplastic cartilage nodules arising from the epiphyseal plate: islands of cartilage and columns of metaphyseal cartilage.^[14]^ According to Mirra et al, dysplastic nodules composed of hypercellular hyaline cartilage become separated from the epiphyseal plate, and subsequently, the settlement process to the metaphysis begins. If this process is short-lasting, islands of cartilage form are recognized. Conversely, if this process is long-lasting, columns of cartilage, which lie from the epiphyseal plate along the metaphyseal area, are seen histologically. The projection of this histological appearance on plain film has been called “fan like septations” or “columnar pattern.” While the island formation is observed in nearly all solitary enchondromas and most specimens of enchondromatosis, “the columnar formation” is virtually pathognomonic for multiple enchondromatosis. However, both ovoid and columnar formations can be seen together in patients with enchondromatosis patients (Fig. 1).

The aim of this study was to demonstrate the clinical and radiological features of 15 patients with a columnar pattern and discuss this rare columnar cartilage pattern apart from the classical form of enchondromas. This pattern is not well defined in the literature and is an obscure phenomenon for most physicians. There is no study on PubMed using the terms “enchondroma” and “columnar pattern” or “fan like.”

## 2. Patients and methods

We included 15 patients who had a enchondroma or enchondromatosis showing columnar pattern on plain radiographs between 1995 and 2020. The diagnosis was established by clinical and radiological examination in all cases. Biopsies were performed in 3 patients (patient numbers 1, 5, and 6) to confirm diagnosis, as the rarity of the lesion can sometimes pose diagnostic challenges. Histological confirmation of normal cartilage provides assurance to the surgeon for conservative patient follow-up without doubts. Additionally, biopsies were performed in 2 patients during surgeries which are curettage and grafting in 1 patient (No: 2) with a large metaphyseal lesion, which posed a risk of fracture and pain, and deformity correction and lengthening surgery in patient No. 10. All biopsy results confirmed benign/normal cartilage.

Enchondromas were classified according Pansuriya et al classification. Characteristics of lesions in terms of their count, location, size, border, internal characteristics and behavior (regression or progression) in the course of the disease during the follow-up period were observed.

All patients underwent X-ray examination of the extremities to check the number of the lesion. Additionally, 9 patients were evaluated by computer tomography (CT) and magnetic resonance imaging (MRI). Two patients had bone scintigraphy in addition to their other radiological examinations. Attenuation values on CT images and signal characteristics of T1W, T2W, and STIR MR images were analyzed.

Age at the initial presentation, gender, enchondromatosis classification, and number of affected physis of the patients are shown in Table 1. The radiologic features of each patient are presented in the following section.

**Table 1:** Age at the initial presentation, gender, affected physis, and enchondromatosis classification of each patient are shown.

No & initials	Age	Sex	Number of affected physis	Classification
1	11.5	Male	1 (Left distal femur)	LE-CP
2	4.7	Male	1 (Left proximal tibia)	LE-CP
3	8.5	Male	1 (left proximal tibia)	LE-CP
4	14.8	Male	1 (Left distal femur)	LE-CP
5	11.8	Male	2 (Right distal femur, proximal tibia)	LE-CP
6	4.6	Male	1 (Left proximal humerus)	LE-CP
7	4.4	Male	2 (Right proximal and distal femur)	LE-CP
8	8.9	Female	3 (Right proximal and distal femur, distal tibia)	LE-CP
9	4.3	Male	Over 10 (Multiple extremities, both side)	Genochondromatosis
10	7.4	Female	4 (Left lower extremity)	ME-CP
11	6.9	Female	4 (Left lower extremity)	ME-CP
12	1.1	Female	6 (Right lover and upper extremity)	ME-CP
13	1.4	Female	8 (Left upper and lower extremity and right proximal fibula)	ME-CP
14	2.9	Male	8 (Left lower and upper extremity including iliac wing)	ME-CP
15	14.3	Female	Over 10 (Right lower and upper extremity including hand)	ME-CP

LE-CP, limited enchondromatosis with columnar pattern; ME-CP, multiple enchondromatosis with columnar pattern.

Five patients had only one affected physis. Eight patients had 3 or less affected physis. The radiological features of the lesions which we defined as “limited enchondromatosis with columnar pattern” were shown in between Figures 2 and 13 (Figs. 2–13). The remaining 7 patients have multiple columns in multiple physis we defined these patient groups as “multiple enchondromatosis with columnar pattern.” Radiological appearances of the 3 out of 7 are shown in Figures 14 to 16 (patient number 9,10,11). These cartilage tissues were partially ossified during the follow-up.

**Figure 2.**
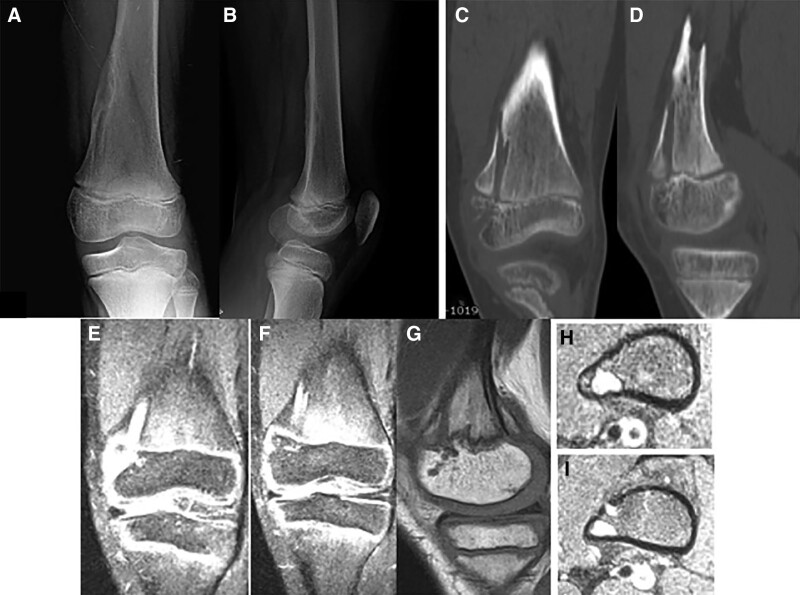
Patient number one presented to the clinic with a slight limb length discrepancy and difficulty walking. His medical history included Charcot–Marie–Tooth disease with bilateral involvement of the foot. Radiological images revealed a single cartilage column on the left distal femur extending from the physis to the metaphyseal area. Biopsy results confirmed a low-grade cartilage tumor. The patient has been under observation for 9 yr. This patient is considered to be in the group of “limited enchondromatosis with columnar pattern.”

**Figure 3.**
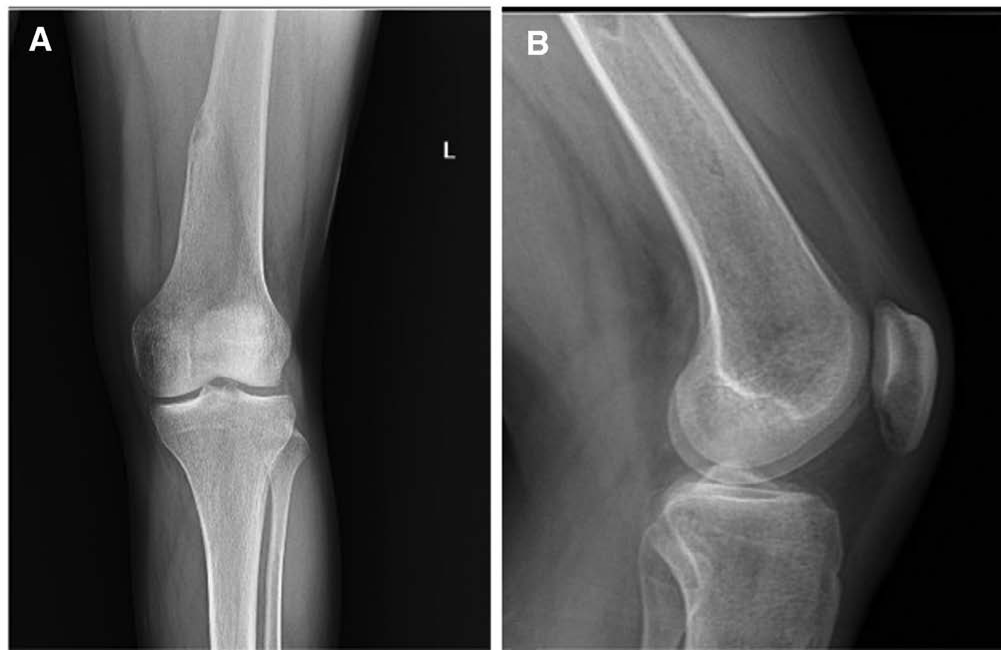
The X-rays of patient No. 1, taken 9 yr after the initial diagnosis, revealed closure of the growth plate, with most of the cartilage tissue having transformed into normal bone by the final follow-up visit at the age of 21 yr old.

**Figure 4.**
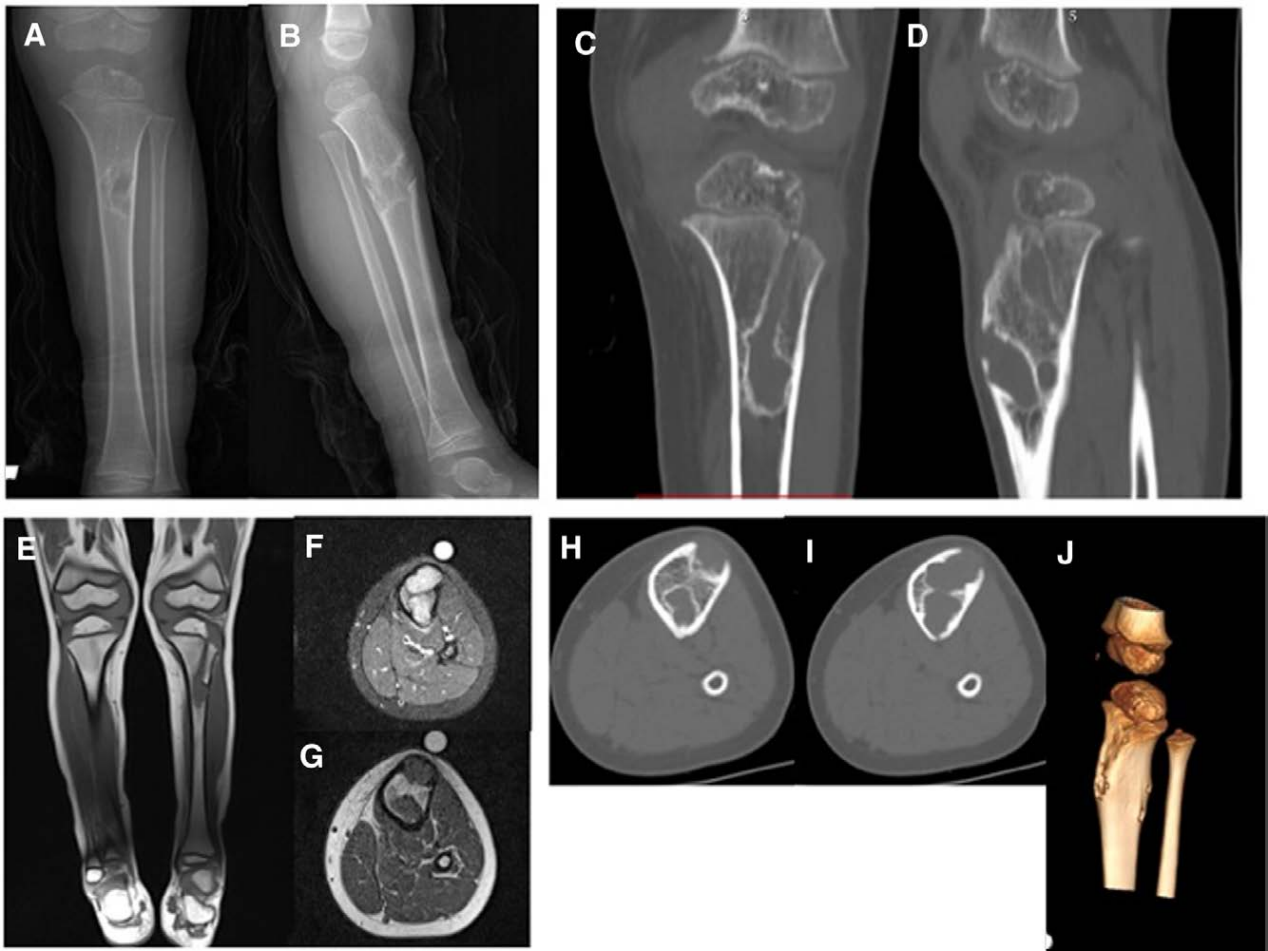
Patient number 2, a 4-yr-old boy, presented to the clinic with leg pain while walking. Radiographic examination revealed a large cartilage column extending from the physis and forming a mass in the proximal diaphysis of the tibia. Due to the risk of fracture, curettage and grafting were performed for the diaphyseal mass. The curated specimen was sent to pathology for analysis, and the pathology report confirmed the presence of normal cartilage. This patient is considered to be in the group of “limited enchondromatosis with columnar pattern.”

**Figure 5.**
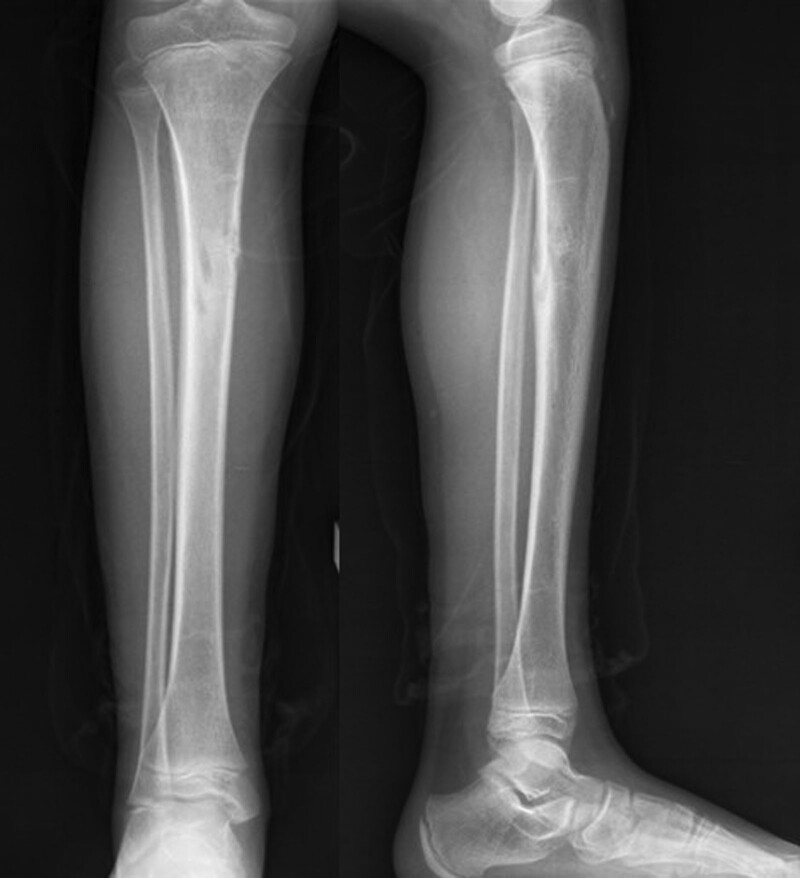
This patient (number 2) was followed up for 8 yr. Final cruris radiographs show partial ossification of the lesions.

**Figure 6.**
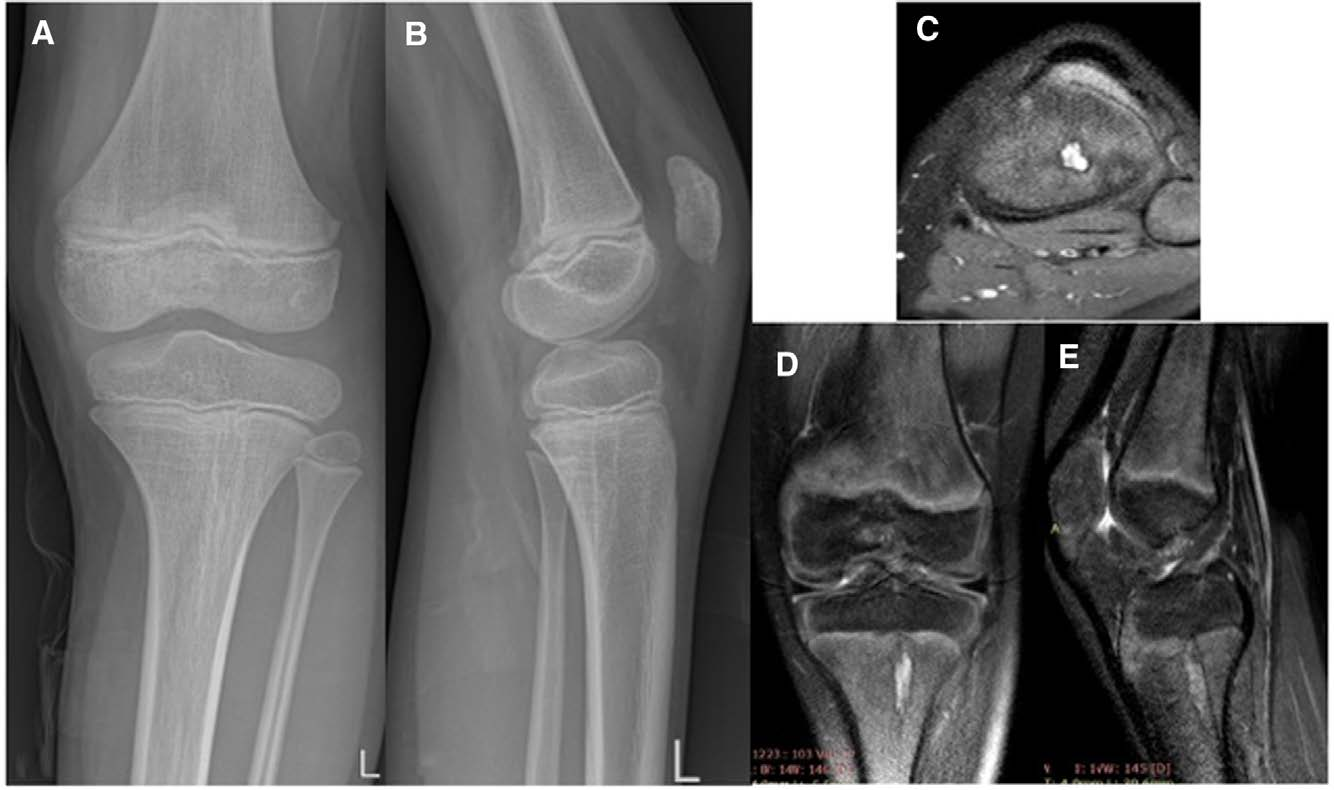
Patient number 3, an 8-yr-old boy, was admitted to the clinic after incidental findings on an X-ray revealed a single cartilage column at the proximal metaphysis of the left tibia. This patient is considered to be in the group of “limited enchondromatosis with columnar pattern.”

**Figure 7.**
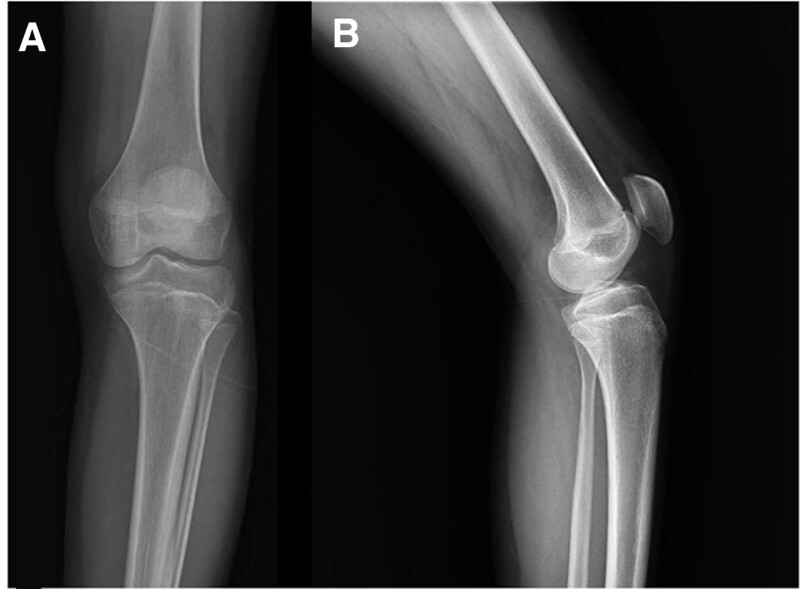
Patient number 3 was conservatively followed up for 7 yr. It was observed that the cartilage column had transformed into normal bone by the age of 15.

**Figure 8.**
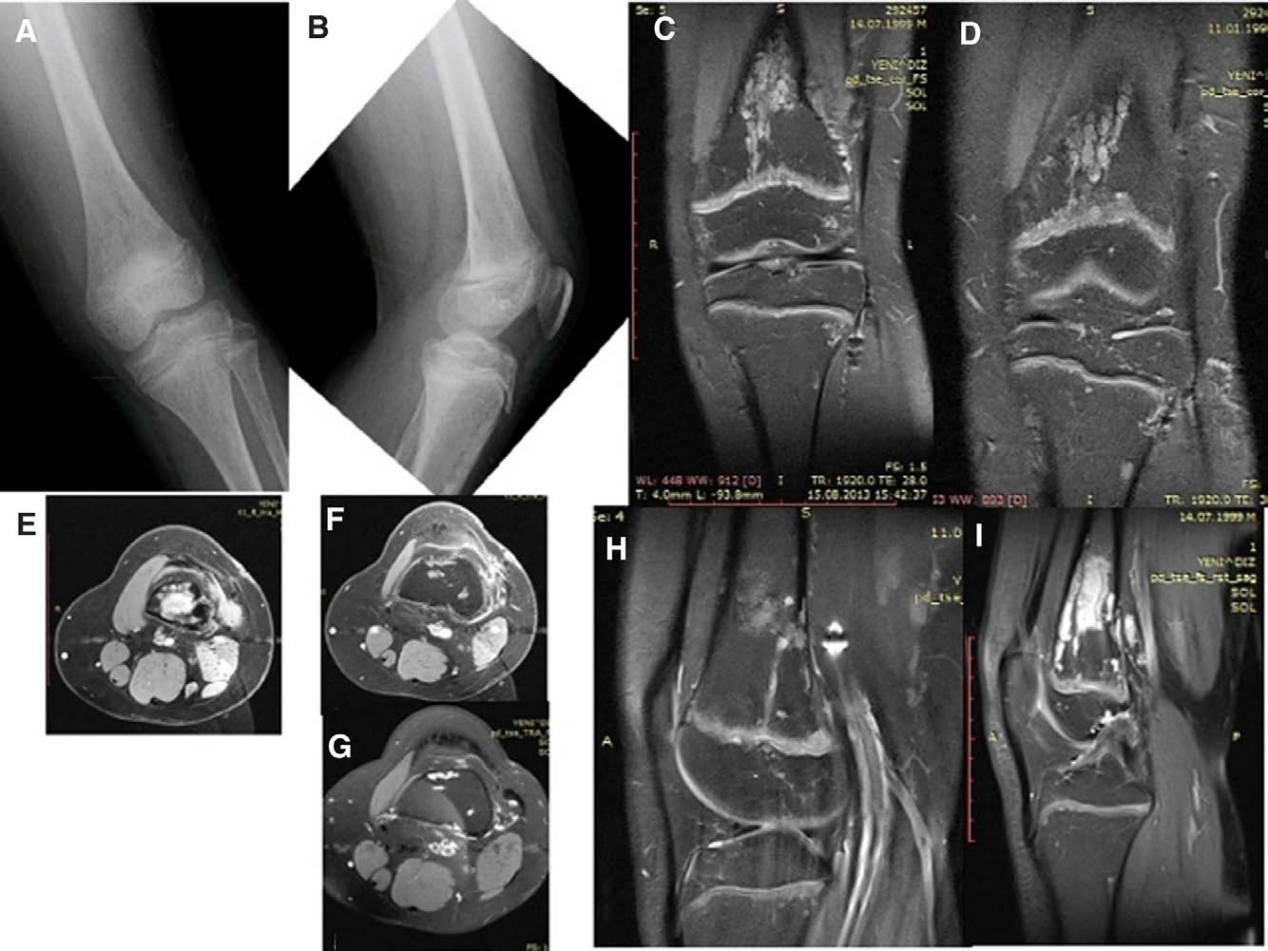
Patient number 4, who has multiple cartilage columns on the distal femoral physis, was conservatively followed up. On the MRI images, the cartilage mass on the metaphyseal area was connected to the physis with the cartilage bridge located anteriorly. This patient is considered to be in the group of “limited enchondromatosis with columnar pattern.”

**Figure 9.**
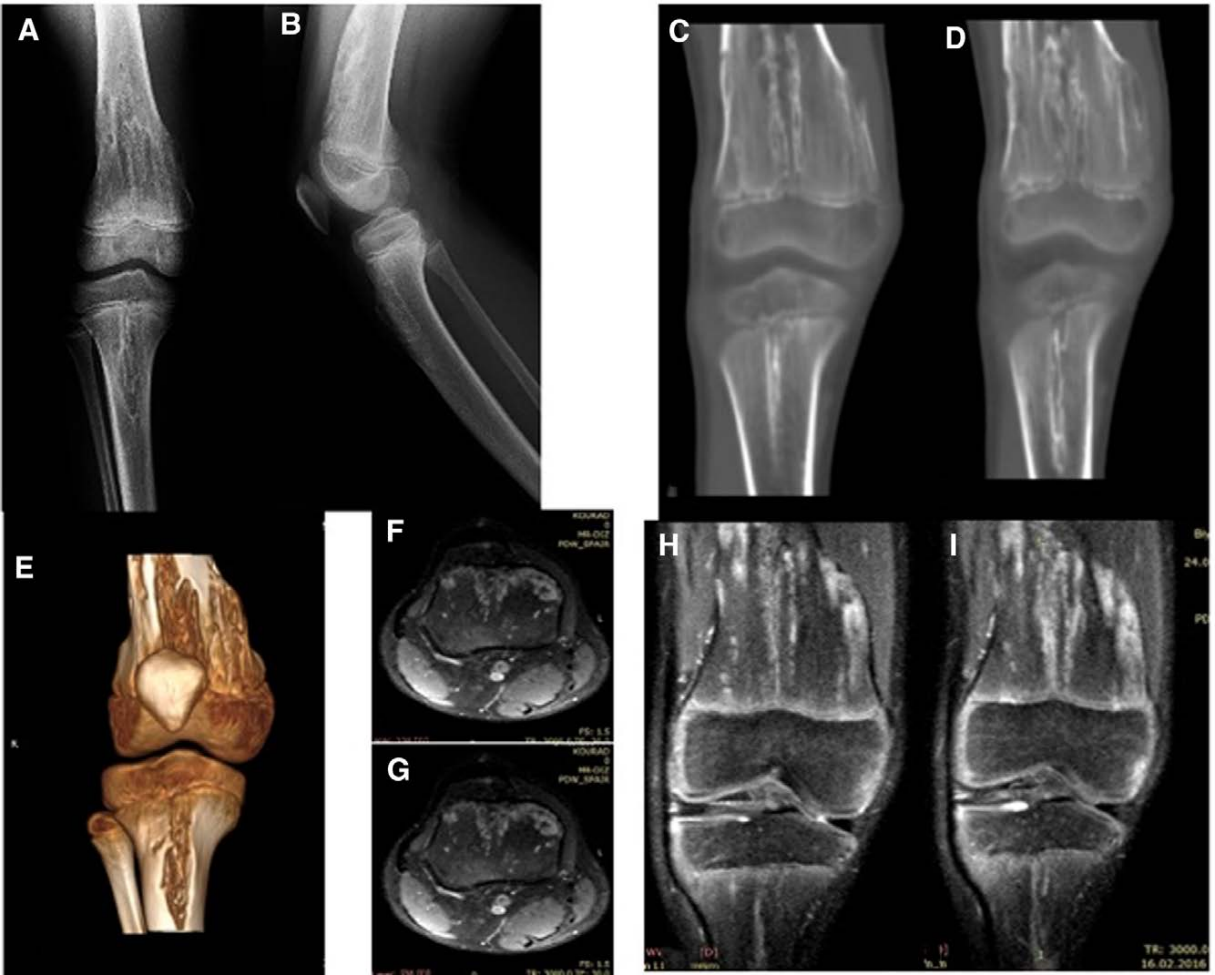
Patient number 5 was referred to our clinic with abnormal knee X-ray, MRI, and CT findings. Multiple columnar patterns were observed at the distal femur and proximal tibia physis. Biopsy results showed benign cartilage tissue, and the patient was conservatively followed up. This patient is considered to be in the group of “limited enchondromatosis with columnar pattern.”

**Figure 10.**
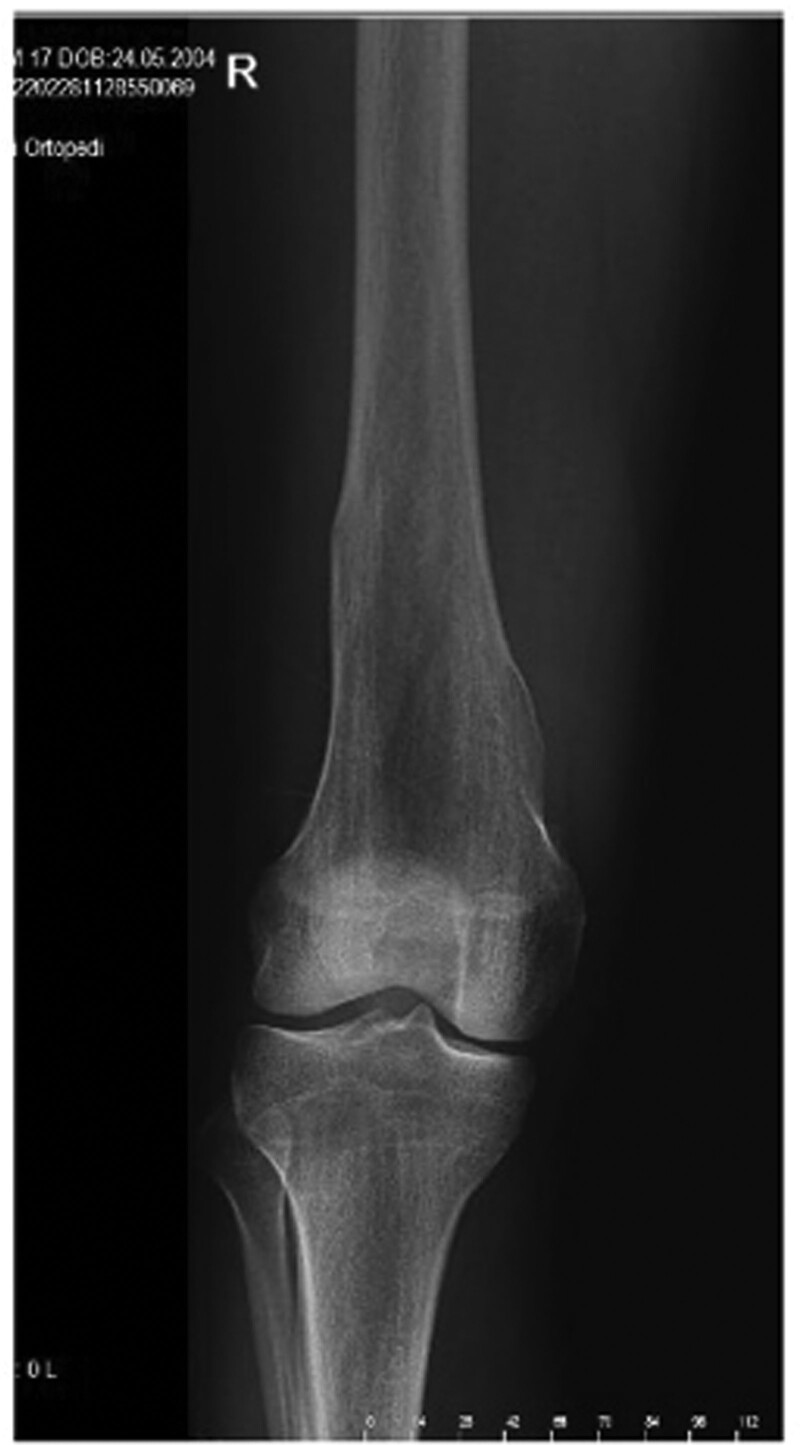
The X-ray of patient number 5 shows that their physis had closed, and all cartilage tissues had transformed into normal bone after 6 yr from the initial admission, at the age of 18 yr old. This is one of the clearest examples of transformation of the prominent cartilage column to the normal bone.

**Figure 11.**
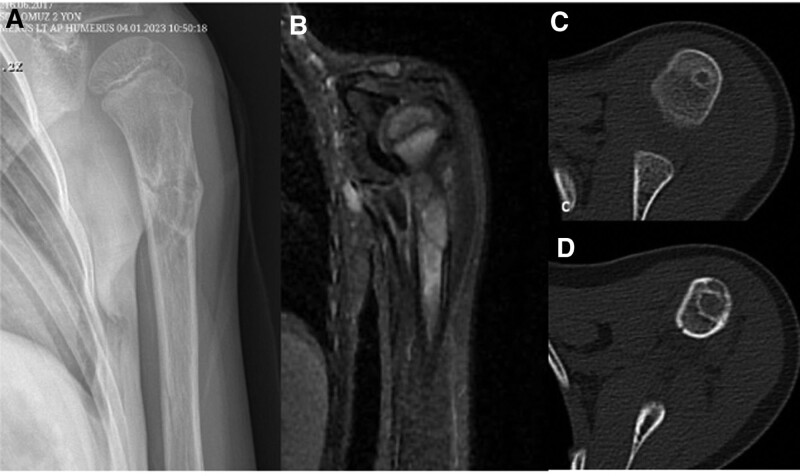
Patient number 6, a 5-yr-old male, exhibited a single large cartilage column on the left proximal humerus. To confirm the diagnosis, a biopsy was performed, and the biopsy results indicated normal cartilage tissue. This patient is being conservatively followed up. This patient is considered to be in the group of “limited enchondromatosis with columnar pattern.”

**Figure 12.**
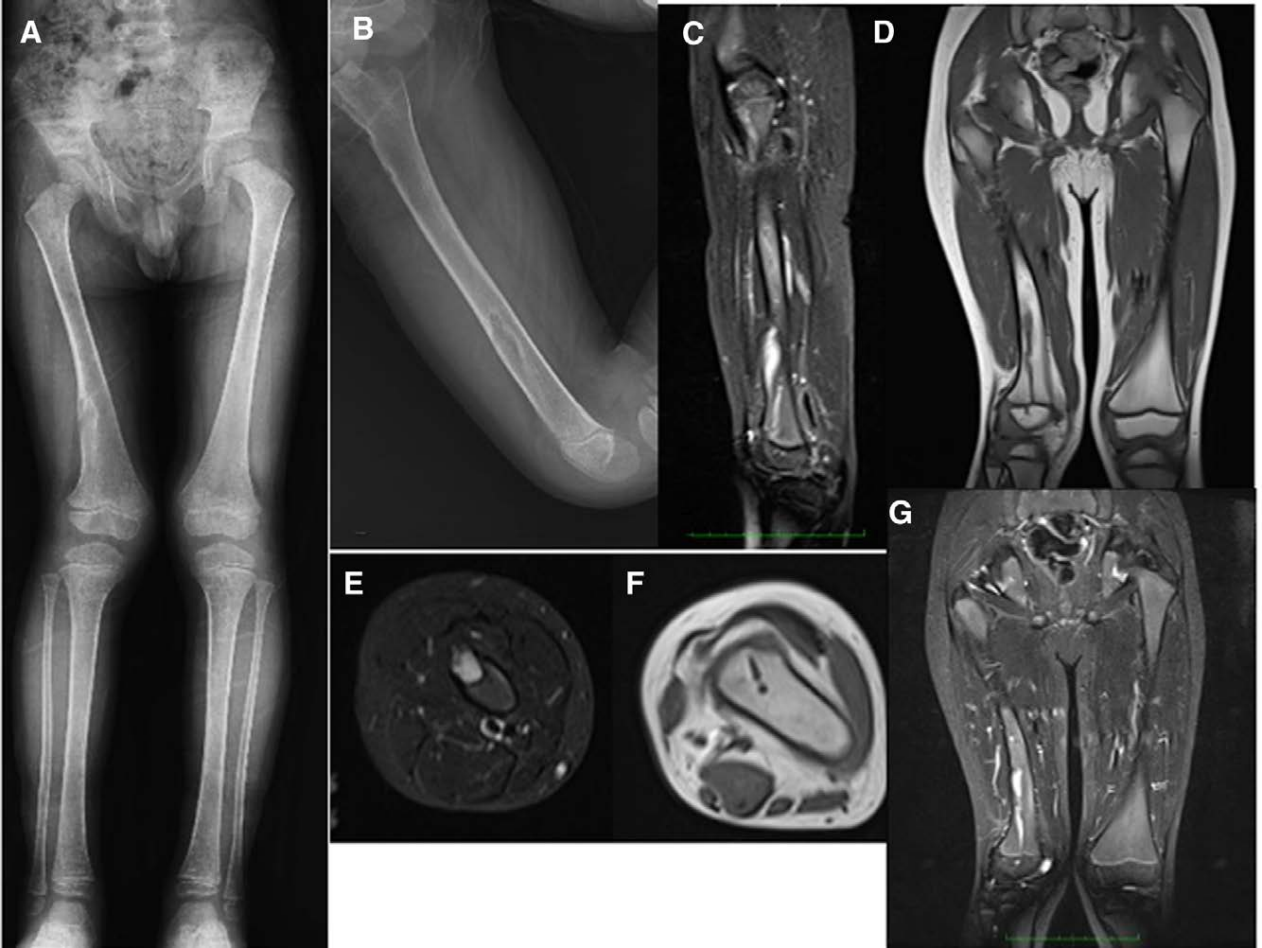
Patient number 7, who presents with a valgus knee, exhibits a distal femur physis that extends into the metaphyseal area, with a single large columnar pattern observed in the metaphysis. Currently, this patient is being conservatively followed up. However, lengthening and deformity correction surgery will be necessary in the future. This patient is considered to be in the group of “limited enchondromatosis with columnar pattern.”

**Figure 13.**
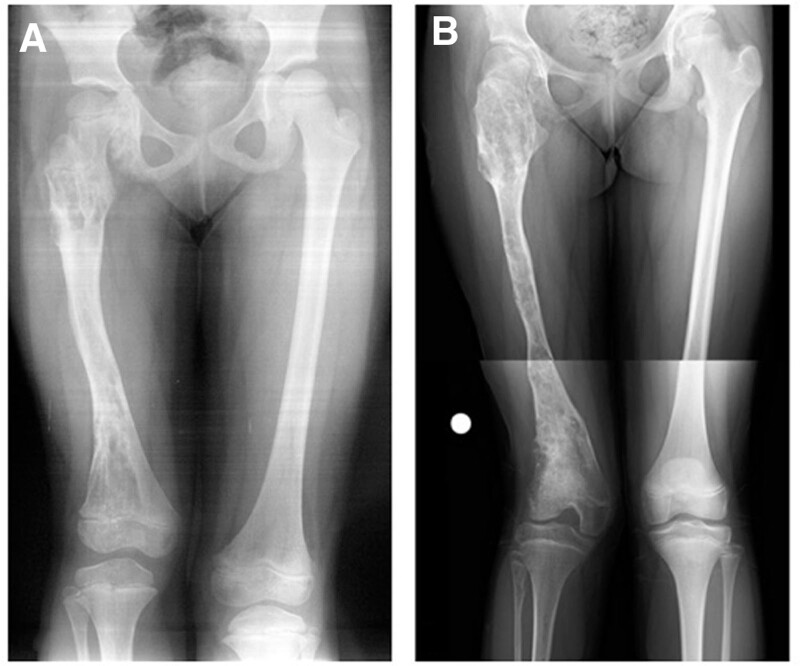
Patient number 8 has 3 affected physes: proximal and distal femur and distal tibia. The appearance of the X-ray at the age of 5 yr old was shown in A. The lesions were partially ossified after 7 yr on X-rays. At the last follow-up, when she was 12 yr old, the physes were still open (B). This patient is considered to be in the group of “limited enchondromatosis with columnar pattern.”

**Figure 14.**
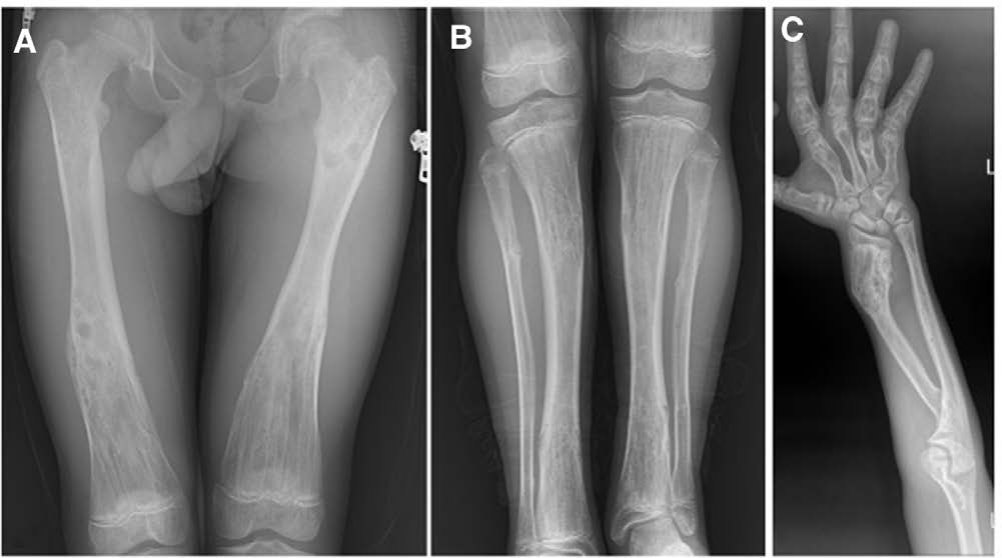
Patient number 9, a 4-yr-old male, underwent genetic evaluation and was diagnosed with genochondromatosis type 2 with an IDH-1 mutation. Radiological evaluation revealed multiple affected physes bilaterally in all 4 extremities. It is a sporadic case with no family history. A longitudinal cartilage column was observed in each metaphyseal area. X-rays obtained at the last control at the age of 11 yr old revealed that the physes were still open (A,B,C). This patient is considered to be in the group of “multiple enchondromatosis with columnar pattern.”

**Figure 15.**
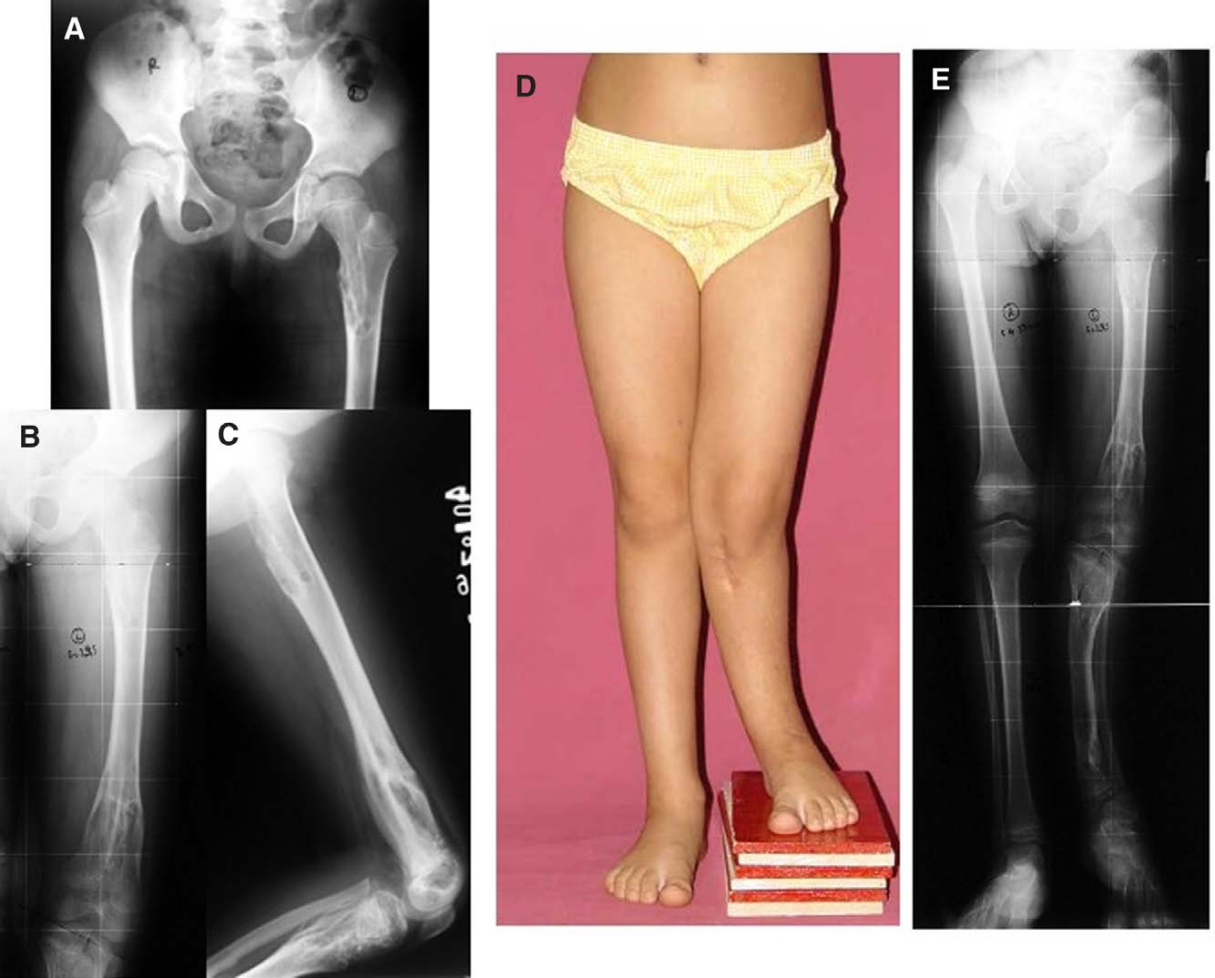
Patient number 10 X-rays reveal multiple affected physis on the left side. Deformity was corrected with multiple operations. This patient is considered to be in the group of “multiple enchondromatosis with columnar pattern.”

**Figure 16.**
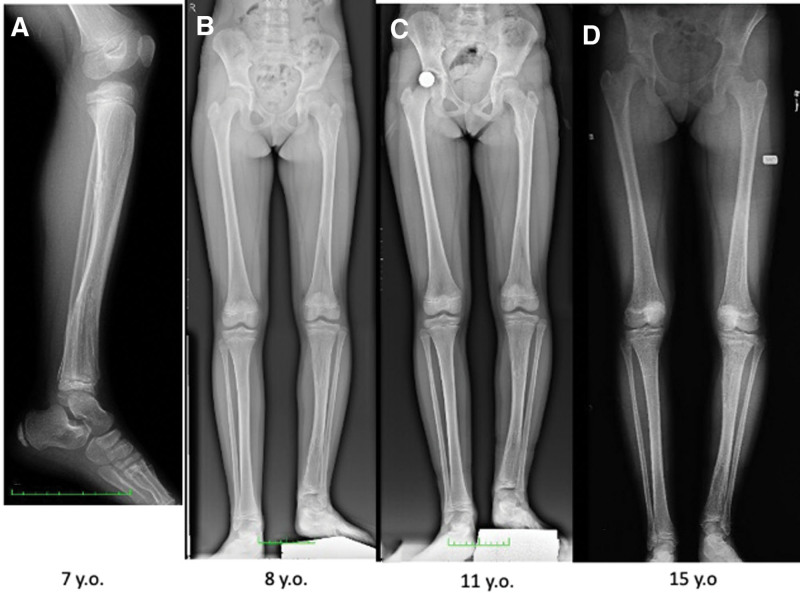
Patient number 11, a female patient, exhibited a columnar pattern seen on multiple affected physis on the left side, with prominence mainly in the tibia physis. Over an 8-yr period, partial ossification was observed. This patient is considered to be in the group of “multiple enchondromatosis with columnar pattern,” and she serves as a good example of multiple affected physes turning into normal cartilage over time.

## 3. Results

The mean follow-up was 62 months (range: 36–96 months). At the initial time of diagnosis, the mean age was 9.7 (range: 4–14) years. Six patients were operated for the deformity or limb reconstruction. The pathologic diagnosis of the 5 patients who underwent biopsy was benign normal cartilage in this cohort. The rest of them followed up conservatively without any malignancy sign.

It is well known that enchondromas can contain calcifications of various shapes (e.g., fine linear, punctate, and popcorn calcification formed by small ring/arc-like densities). We have not seen calcifications in the enchondromas showing columnar pattern.

**Figure 17.**
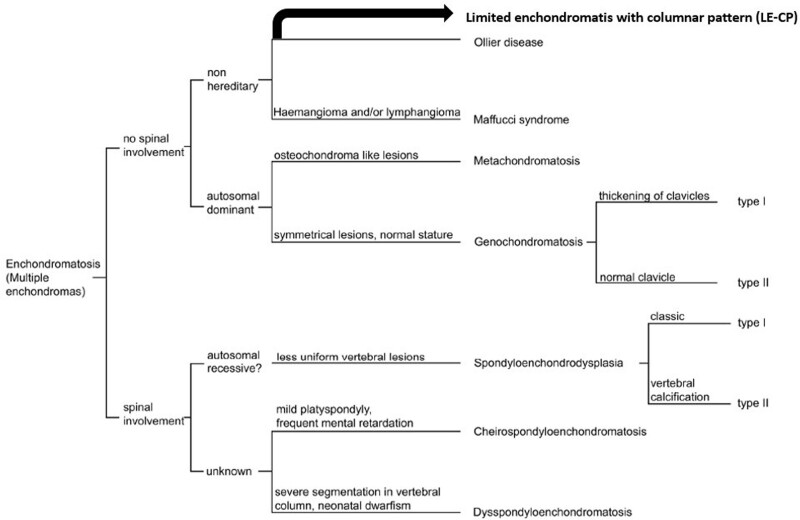
Enchondromatosis classification, as cited from the study conducted by Pansuriya et al^[15]^ with our modification indicated by the black arrow.

All lesions arise in one side of the body except for the bilateral genochondromatosis case (patient 9). Five patients have only one affected physis. Eight patients had 3 or less affected physis. We reclassified these patients’ group who had up to 3 affected physis as limited enchondromatosis with a columnar pattern (LE-CP) (Fig. 17).

## 4. Discussion

After the first description of the multiple enchondromatosis in 1898 by the French surgeon Ollier, classification of the multiple enchondromatosis was enhanced by the studies of the Spranger and Pansuriya et al.^[10, 15–18]^ Our cases were classified using the Pansuriya et al classification system. Additionally, one more bullet was added to this classification system named as “limited enchondromatosis with columnar pattern,” which showed as black arrow on the illustration (Fig. 17).^[15]^

Multiple enchondromatosis is easy to recognize due to its prominent clinical and radiological findings. However, patients with a solitary cartilage lesion, particularly those with a columnar cartilage pattern, may not present with clinical signs. Additionally, we do not know the exact prevalence of columnar pattern because of these lesions turn into normal bone with the endochondral ossification at the end of growth, similar to the ossification process in the physeal plate. Because of these reasons, the actual prevalence of the physis abnormalities may be higher than estimated. However, this argument needs to be confirmed with further studies involving a larger number of patients, which may be difficult due to the rarity of the condition.

We observed that in most of patient with the limited enchondromatosis with columnar pattern, lesions turn in to normal bone like physis. We believe that “limited enchondromatosis with columnar pattern” reflect a developmental anomaly of the cartilage tissue rather than a true neoplasia. Orthopedic surgeons and radiologists should be aware of recognizing cartilage columns and knowing the imaging characteristics of lesions to prevent unnecessary invasive procedures. Chronic osteomyelitis, osteochondroma, and fibrous dysplasia can be considered in the radiological differential diagnosis. Clinical and radiological findings, especially the relationship of the lesion with the physeal cartilage, could help making correct diagnosis.

Based on our observations, the columnar pattern is a benign abnormality of the physis, with most cases eventually transforming into normal bone. However, there is a risk of malignant transformation, with approximately 30% developing into chondrosarcoma and 50% associated with any malignancy in Ollier and Maffucci syndromes. Physicians should be aware of this risk and conduct regular surveillance using appropriate radiologic modalities. For children with Ollier disease, periodic clinical screenings every 6 to 12 months and plain radiographs every 2 to 3 years are recommended to detect growth abnormalities early for surgical treatment. In adults, regular clinical examinations every 12 to 24 months and plain radiographs every 2 to 3 years, depending on the location of the enchondroma, help detect and treat malignant transformations promptly. Starting at 25 years of age, individuals should undergo a thorough clinical examination along with a full-body MRI every other year due to the risk of chondrosarcoma, brain tumors, and other malignancies.^[19, 20]^ MRI findings such as bone edema, periosteal reaction, and soft tissue edema may indicate a high-grade malignant transformation of chondral tumors in patients with enchondromatosis.^[21]^

This study has limitations. Patients followed up prospectively, however some of the patients still growing with open physis. The follows up of these patients is not ended. Our hospital is a tertiary health center that admits bone and soft tissue tumor cases from different parts of the country for advanced diagnosis and treatment options. As a result, patients undergo radiological examinations on the different vendors and models of CT and MRI scanners from several hospitals. T1-weighted, T2-weighted, short-tau inversion recovery (STIR) images were obtained on different MR scanners with varying TR, TE, field of view, and matrix. Thus, the imaging protocols and parameters are not uniform.

In conclusion, physeal developmental abnormality may manifest in a variety of appearances. According to our observations, the columnar pattern is part of physeal disorganization, and it may occur in a single column on a physis or multiple columns on multiple physis.

“Limited enchondromatosis with columnar pattern” and related physis act as normal endochondral ossification process, and surgery is unnecessary unless to there is a risk of fracture or severe deformity. Further awareness of this unique subset of patients may increase our understanding of the disease and improve patient outcomes.

## Author contributions

**Conceptualization:** Ahmet Salduz, Serkan Bayram.

**Data curation:** Ahmet Salduz, Serkan Bayram, Mesut Bulakçi.

**Investigation:** Serkan Bayram, Mesut Bulakçi.

**Methodology:** Ahmet Salduz, Serkan Bayram, Mesut Bulakçi.

**Resources:** Serkan Bayram.

**Supervision:** Serkan Bayram.

**Writing – original draft:** Ahmet Salduz.

**Writing – review & editing:** Mesut Bulakçi.
